# Supplementary figures and images for: Calibration of parameters in microscopic traffic flow simulation models considering micro-meteorological information
Source: PLoS One. 2025 Jul 7;20(7):e0326191. doi: 10.1371/journal.pone.0326191 (PMC12233247; doi:10.1371/journal.pone.0326191)

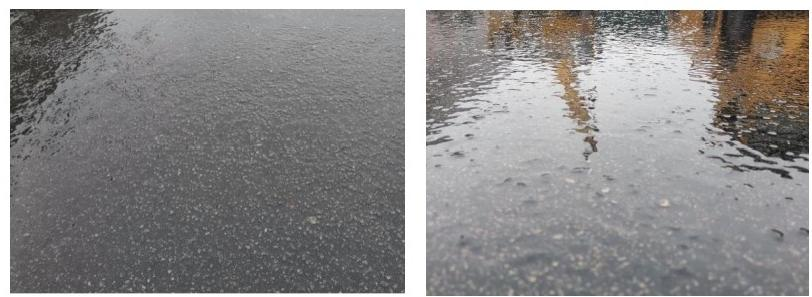

Supplement: S1 File — (ZIP) [file pone.0326191.s002.zip › Original Files/Fig 1. Light rain(a)and heavy rain(b) film thickness under meteorological conditions.png]

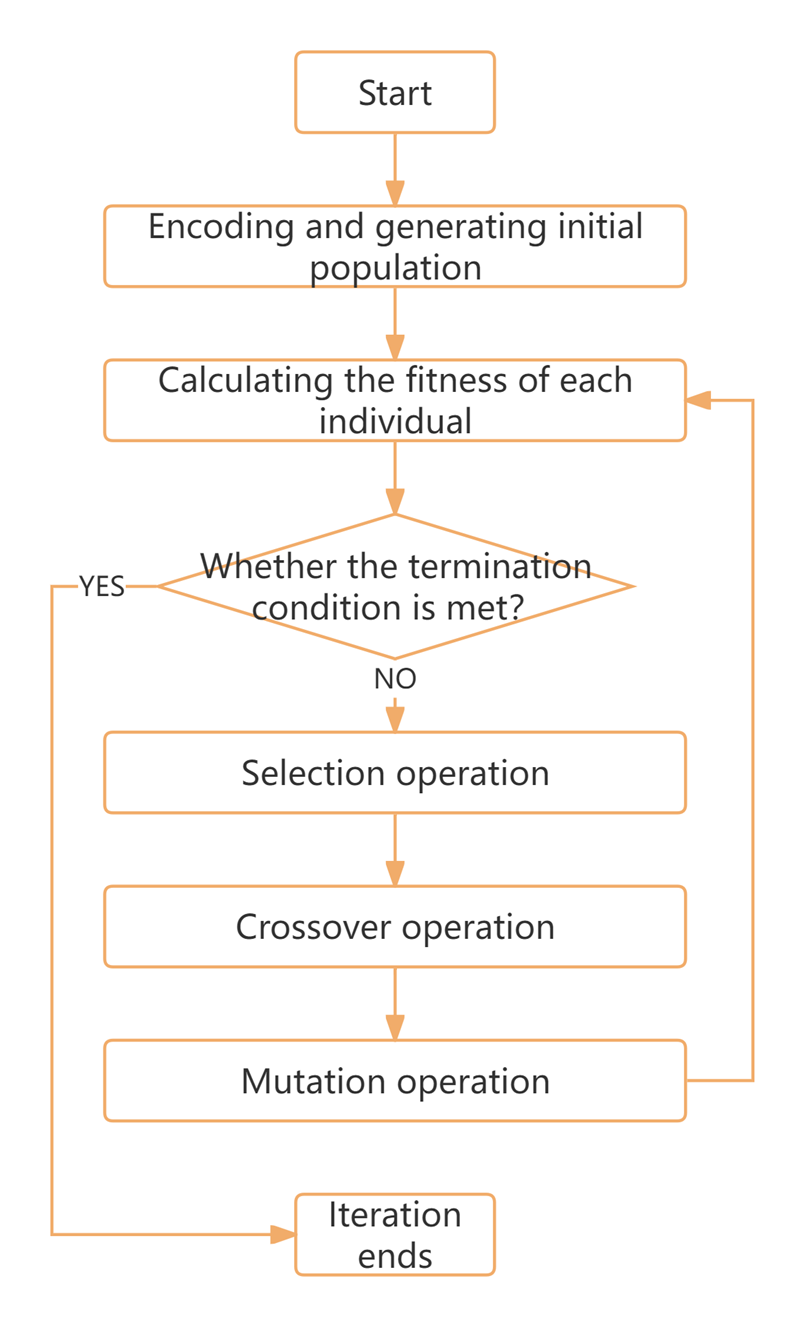

Supplement: S1 File — (ZIP) [file pone.0326191.s002.zip › Original Files/Fig 10. Flowchart of the particle swarm optimization algorithm.png]

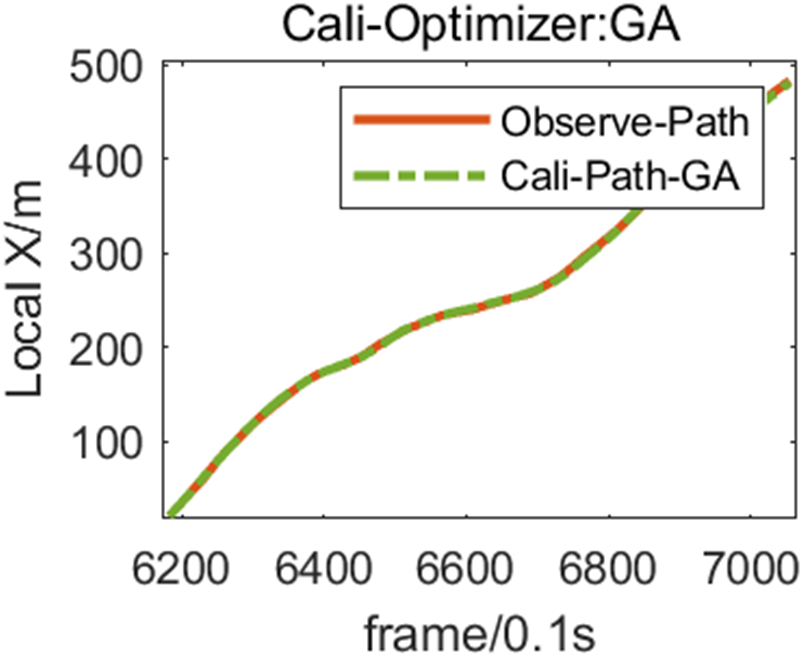

Supplement: S1 File — (ZIP) [file pone.0326191.s002.zip › Original Files/Fig 11-1. Comparison of following vehicle conditions before and after I-Wiedemann99 parameter calibration.png]

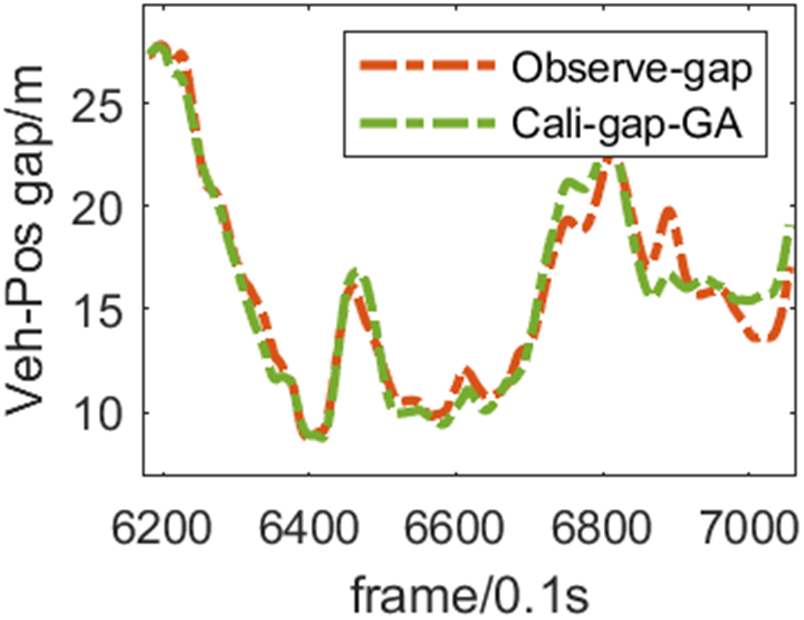

Supplement: S1 File — (ZIP) [file pone.0326191.s002.zip › Original Files/Fig 11-2. Comparison of following vehicle conditions before and after I-Wiedemann99 parameter calibration.png]

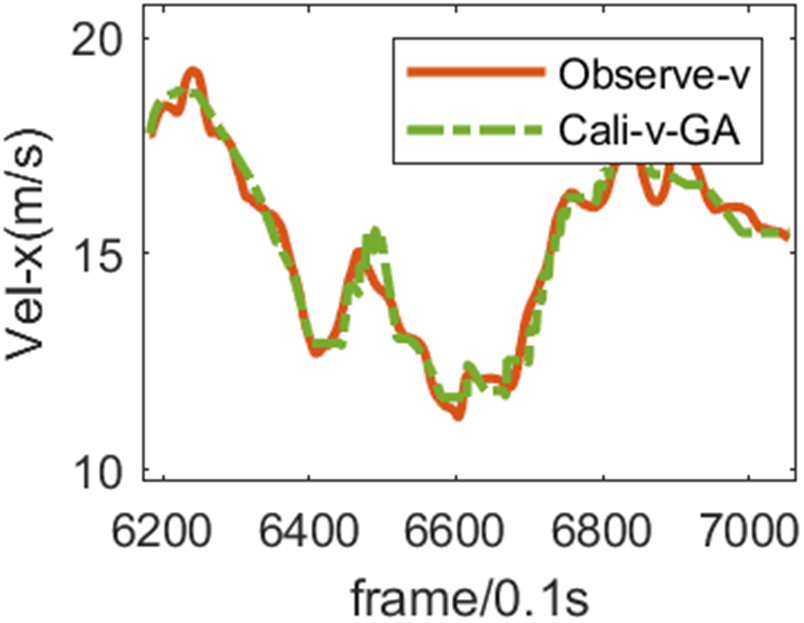

Supplement: S1 File — (ZIP) [file pone.0326191.s002.zip › Original Files/Fig 11-3. Comparison of following vehicle conditions before and after I-Wiedemann99 parameter calibration.png]

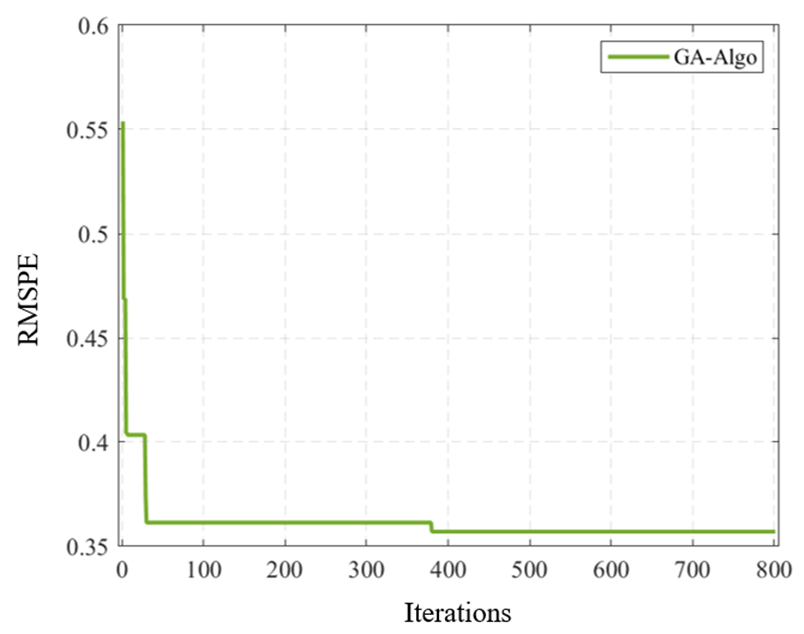

Supplement: S1 File — (ZIP) [file pone.0326191.s002.zip › Original Files/Fig 12. E-RMSPE variation curve for I-Wiedemann99 model parameter calibration.png]

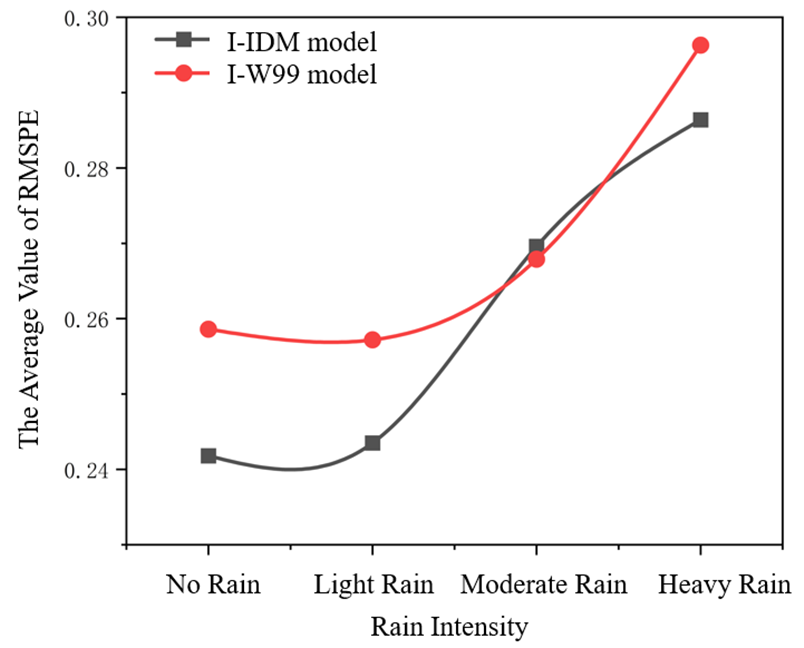

Supplement: S1 File — (ZIP) [file pone.0326191.s002.zip › Original Files/Fig 13. Variation of the average validation error in following vehicle model calibration.png]

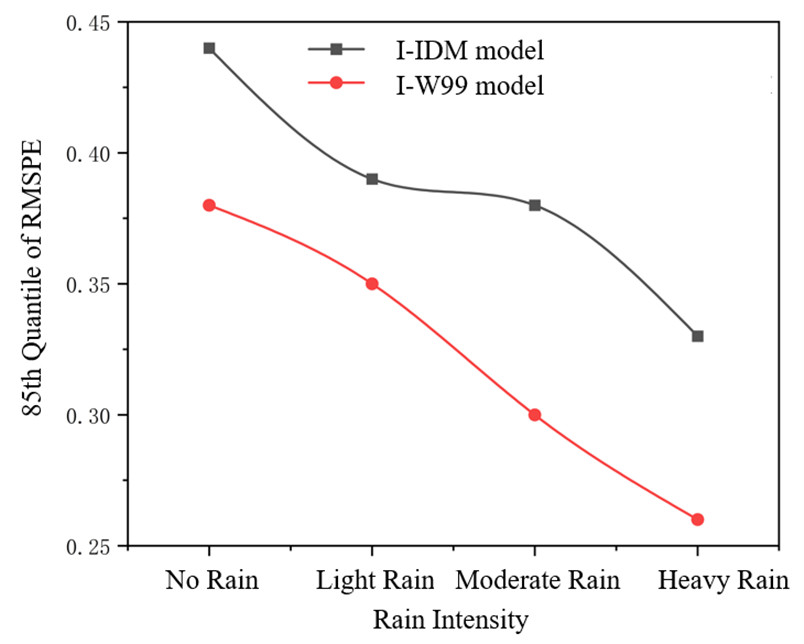

Supplement: S1 File — (ZIP) [file pone.0326191.s002.zip › Original Files/Fig 14. Variation curve of the 85th percentile RMSPE for I-IDM and I-Wiedemann99 under different rain intensity levels.png]

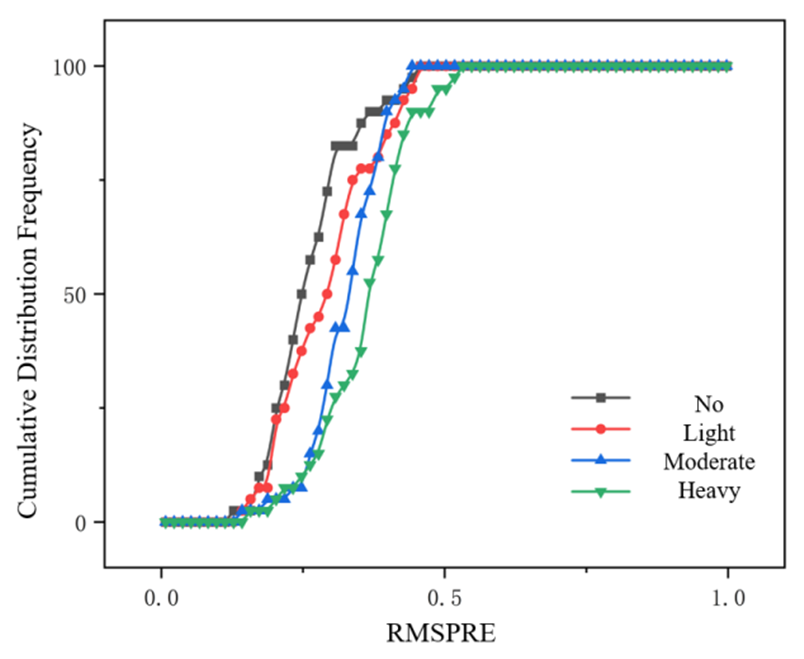

Supplement: S1 File — (ZIP) [file pone.0326191.s002.zip › Original Files/Fig 15. Cumulative frequency distribution of I-IDM model under different rainfall intensities.png]

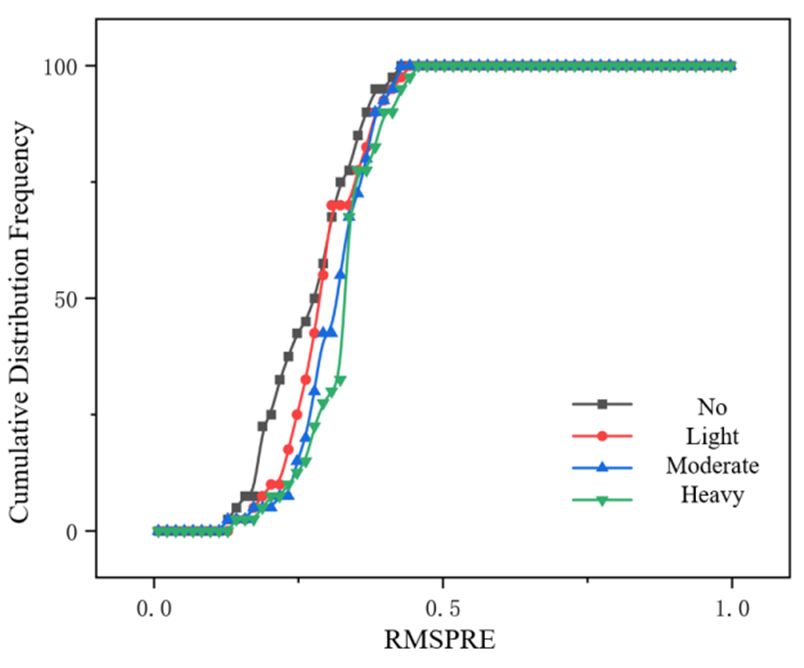

Supplement: S1 File — (ZIP) [file pone.0326191.s002.zip › Original Files/Fig 16. Cumulative frequency distribution of I-Wiedemann99 model under different rainfall intensities.png]

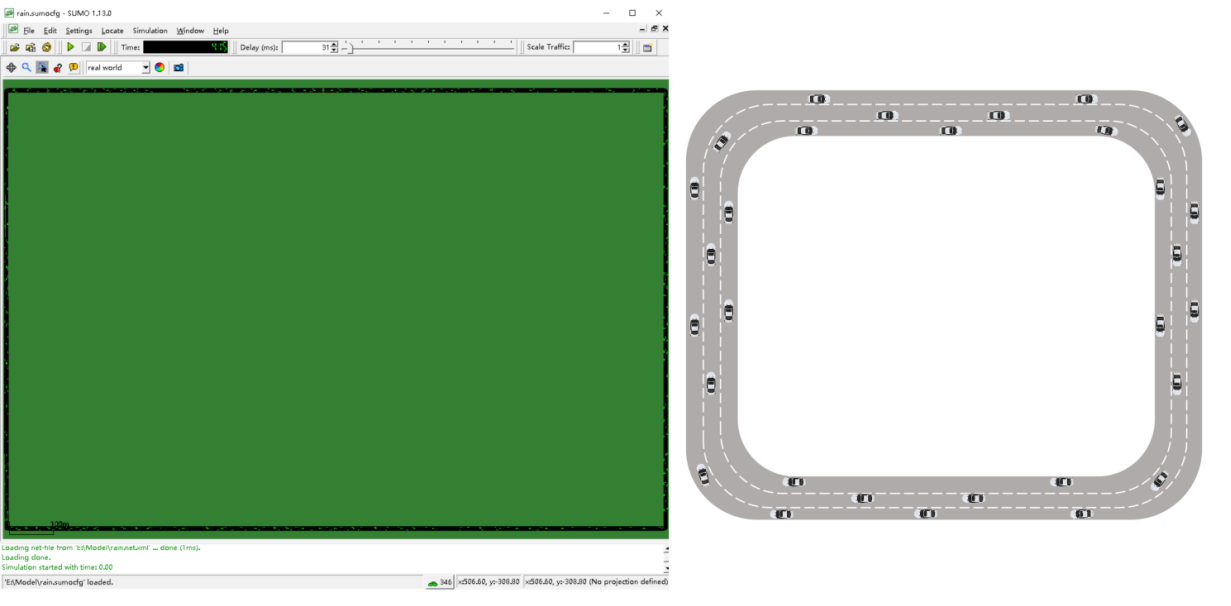

Supplement: S1 File — (ZIP) [file pone.0326191.s002.zip › Original Files/Fig 17. Schematic diagram of the closed-loop simulation scenario.png]

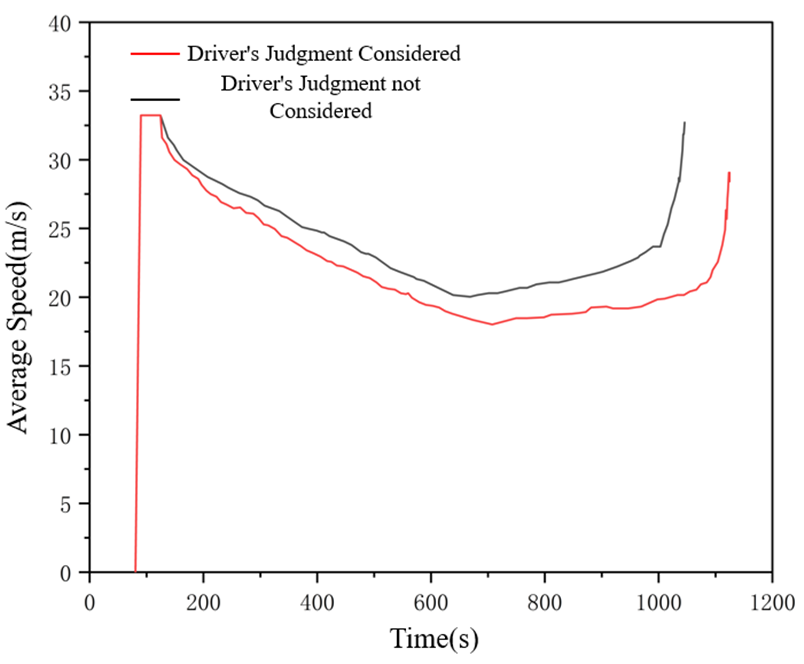

Supplement: S1 File — (ZIP) [file pone.0326191.s002.zip › Original Files/Fig 18. Influence of drivers judgment of road conditions under micrometeorological conditions on average road speed.png]

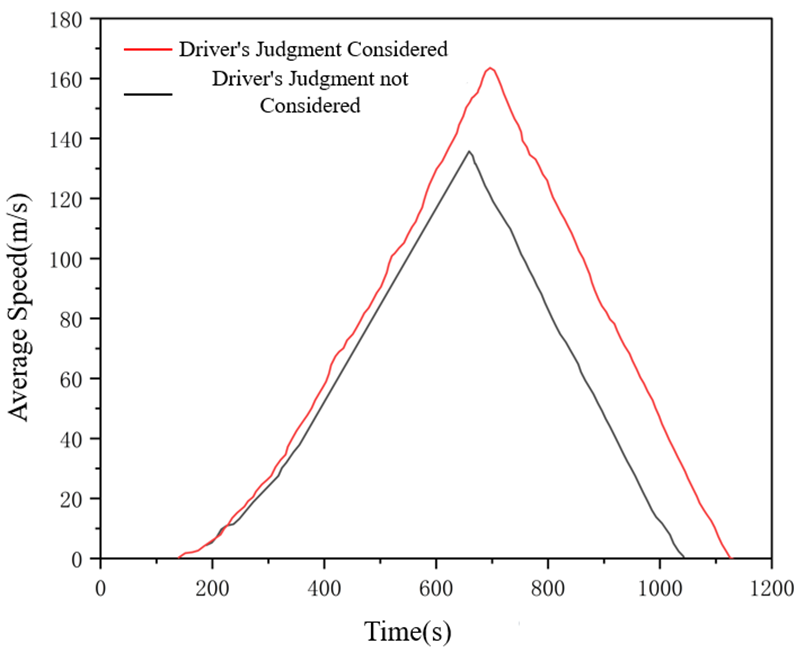

Supplement: S1 File — (ZIP) [file pone.0326191.s002.zip › Original Files/Fig 19. Influence of drivers judgment of road conditions under the influence of micrometeorological conditions on average road loss time.png]

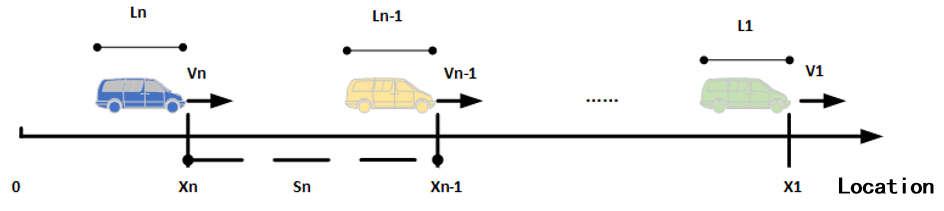

Supplement: S1 File — (ZIP) [file pone.0326191.s002.zip › Original Files/Fig 2. Schematic diagram of IDM model following motion.png]

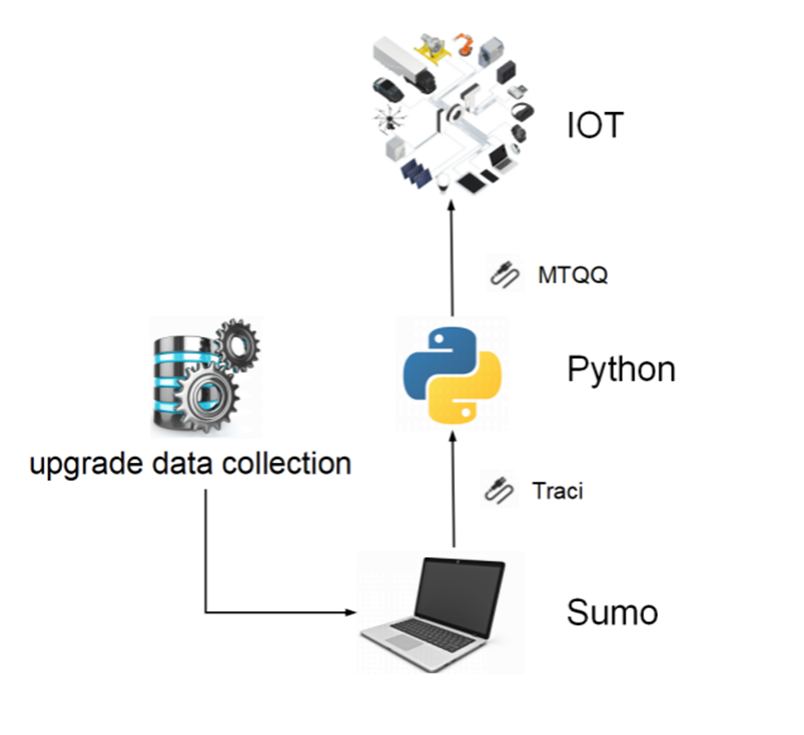

Supplement: S1 File — (ZIP) [file pone.0326191.s002.zip › Original Files/Fig 20. The calibration methodology integrated into practices.png]

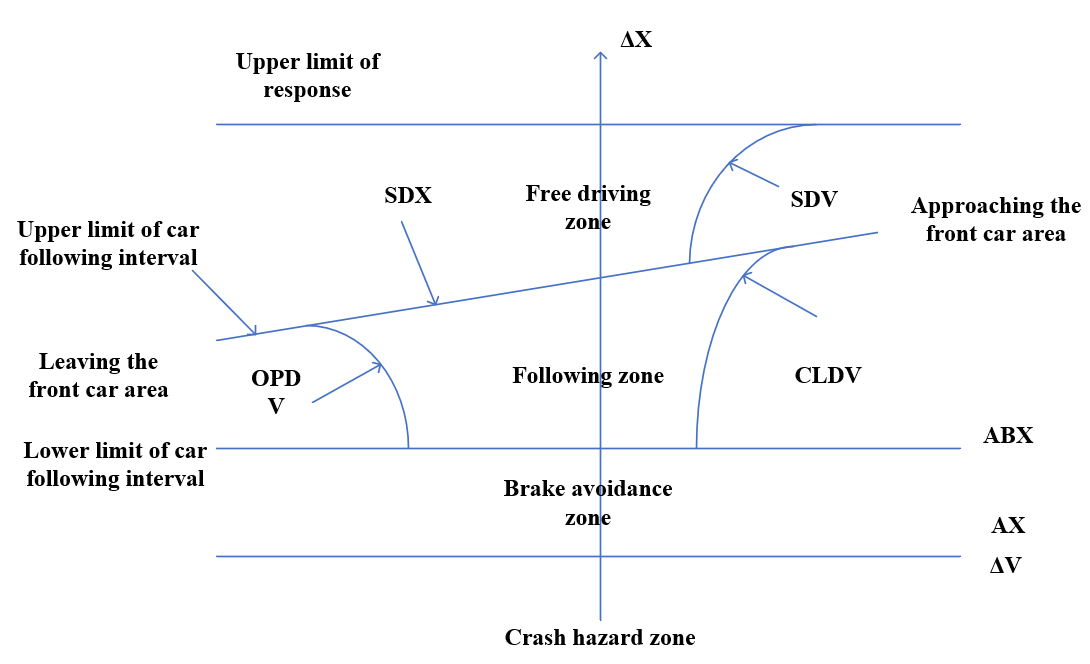

Supplement: S1 File — (ZIP) [file pone.0326191.s002.zip › Original Files/Fig 3. Schematic diagram of the Wiedemann99 model fol lowing motion.png]

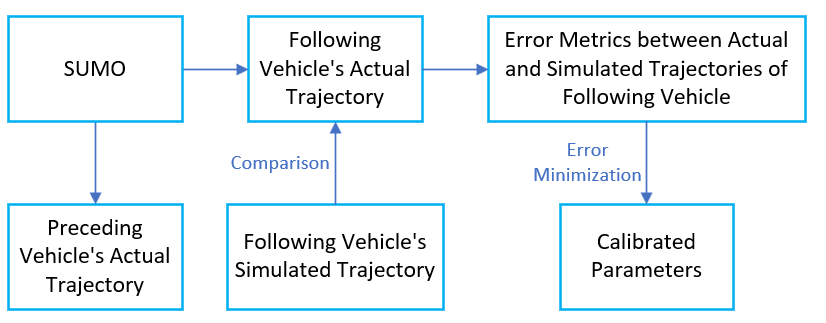

Supplement: S1 File — (ZIP) [file pone.0326191.s002.zip › Original Files/Fig 4. Following model parameter calibration flow chart.png]

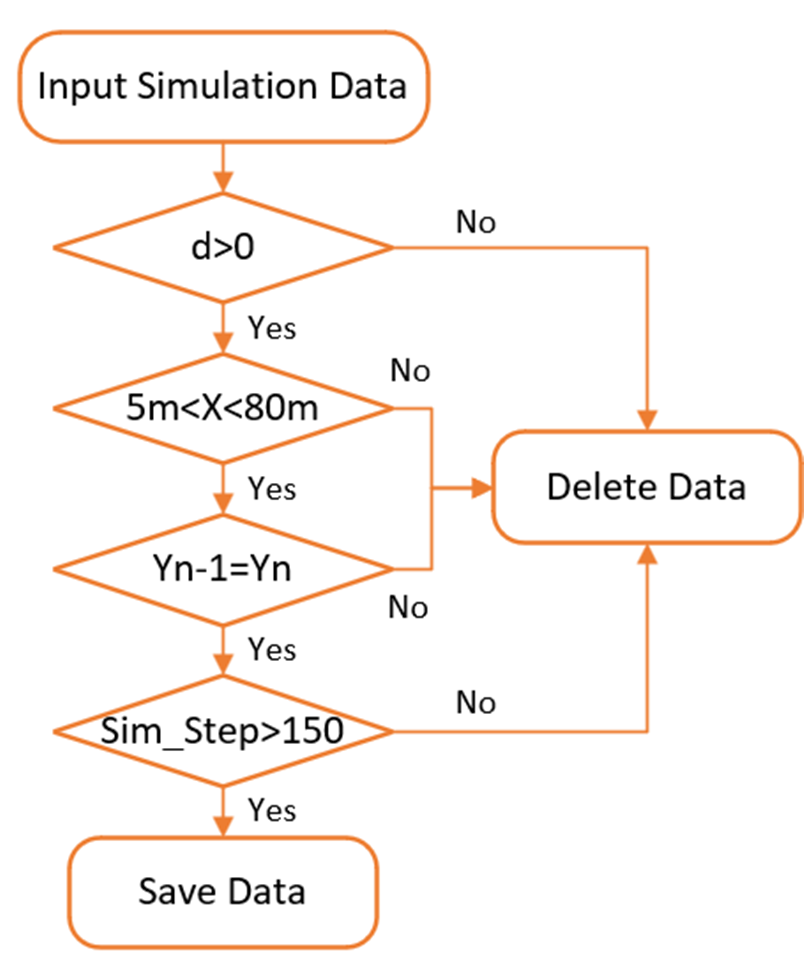

Supplement: S1 File — (ZIP) [file pone.0326191.s002.zip › Original Files/Fig 5. Process of extracting follow events.png]

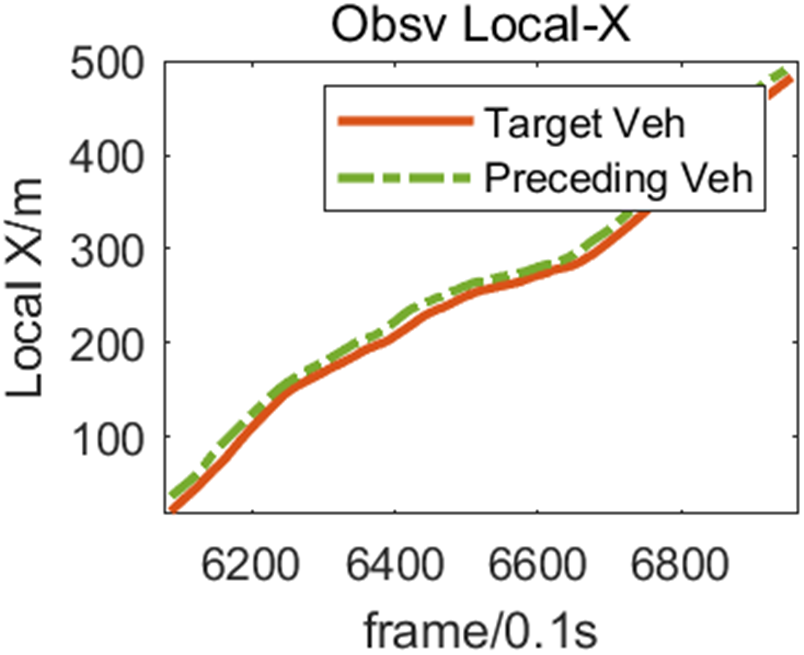

Supplement: S1 File — (ZIP) [file pone.0326191.s002.zip › Original Files/Fig 6-1. Example vehicle following event.png]

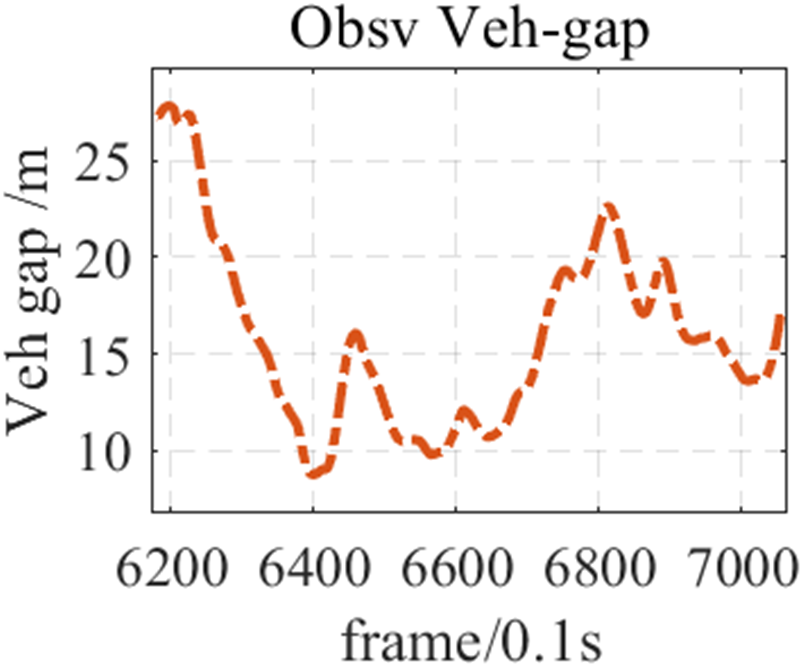

Supplement: S1 File — (ZIP) [file pone.0326191.s002.zip › Original Files/Fig 6-2. Example vehicle following event.png]

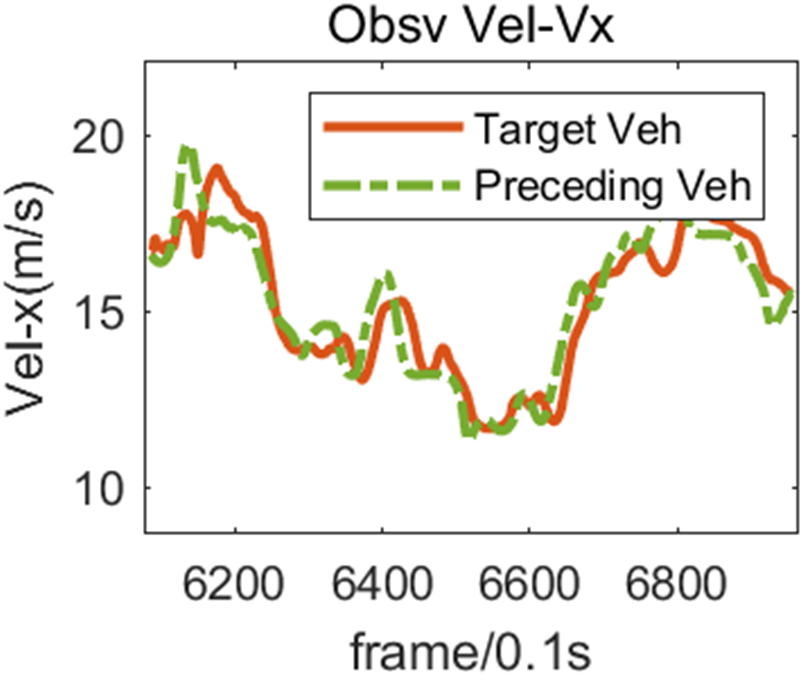

Supplement: S1 File — (ZIP) [file pone.0326191.s002.zip › Original Files/Fig 6-3. Example vehicle following event.png]

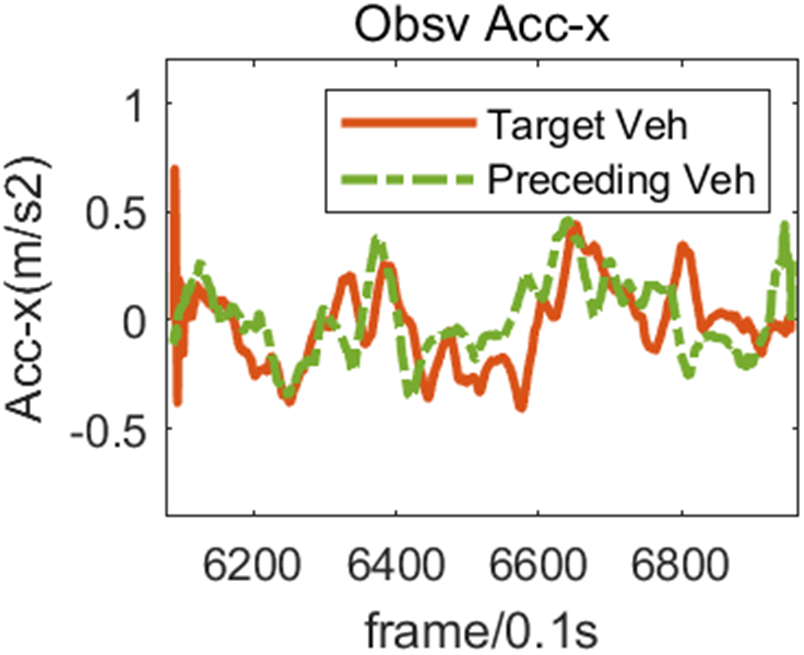

Supplement: S1 File — (ZIP) [file pone.0326191.s002.zip › Original Files/Fig 6-4. Example vehicle following event.png]

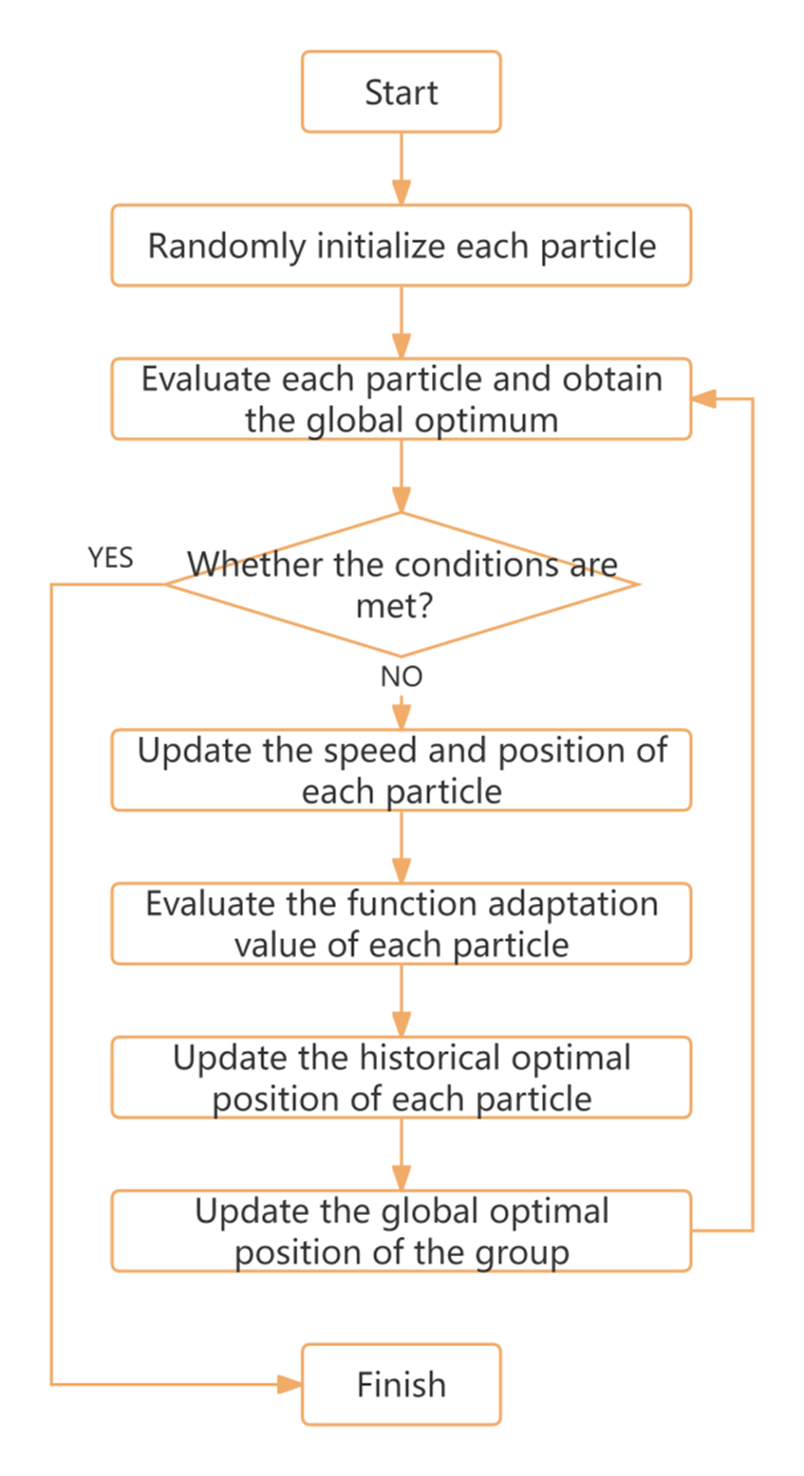

Supplement: S1 File — (ZIP) [file pone.0326191.s002.zip › Original Files/Fig 7. Flowchart of the particle swarm optimization algorithm.png]

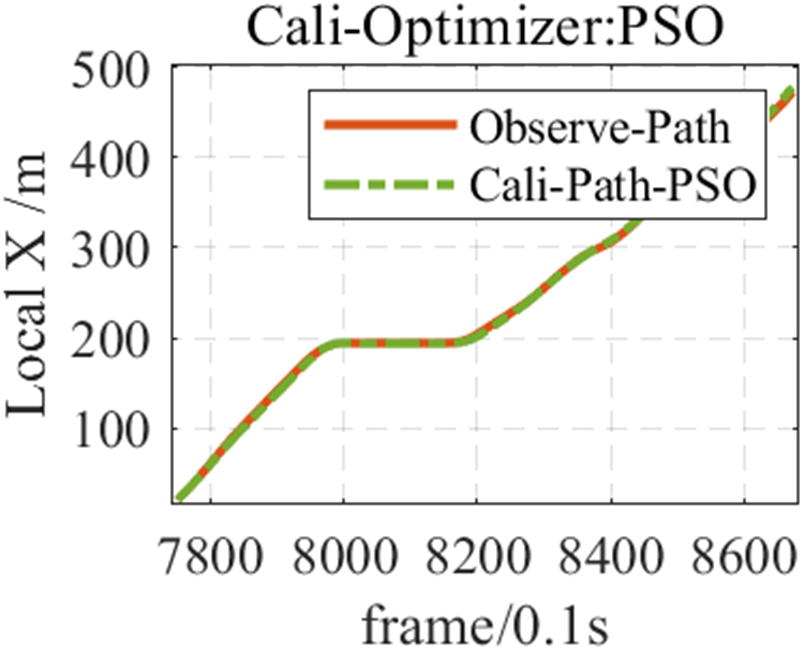

Supplement: S1 File — (ZIP) [file pone.0326191.s002.zip › Original Files/Fig 8-1. Comparison of I-IDM parameters after calibration and before and after calibration.png]

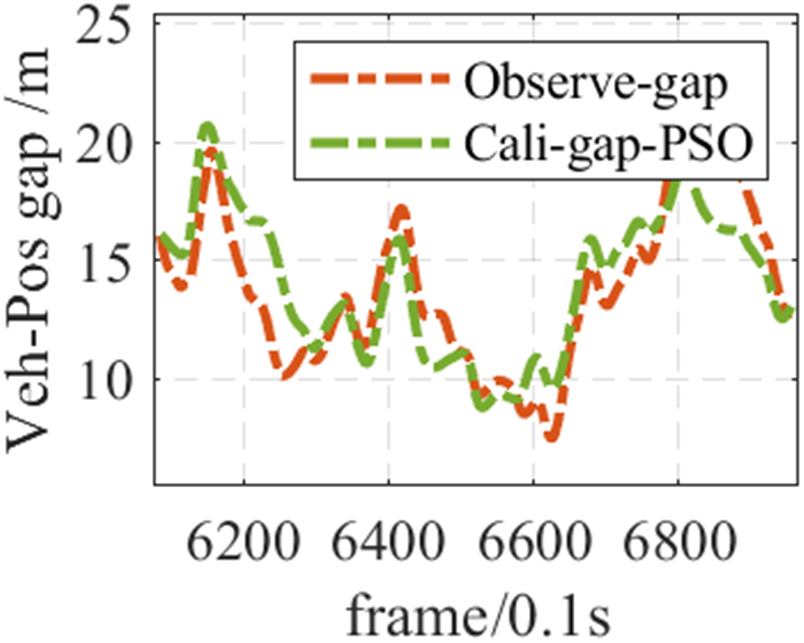

Supplement: S1 File — (ZIP) [file pone.0326191.s002.zip › Original Files/Fig 8-2. Comparison of I-IDM parameters after calibration and before and after calibration.png]

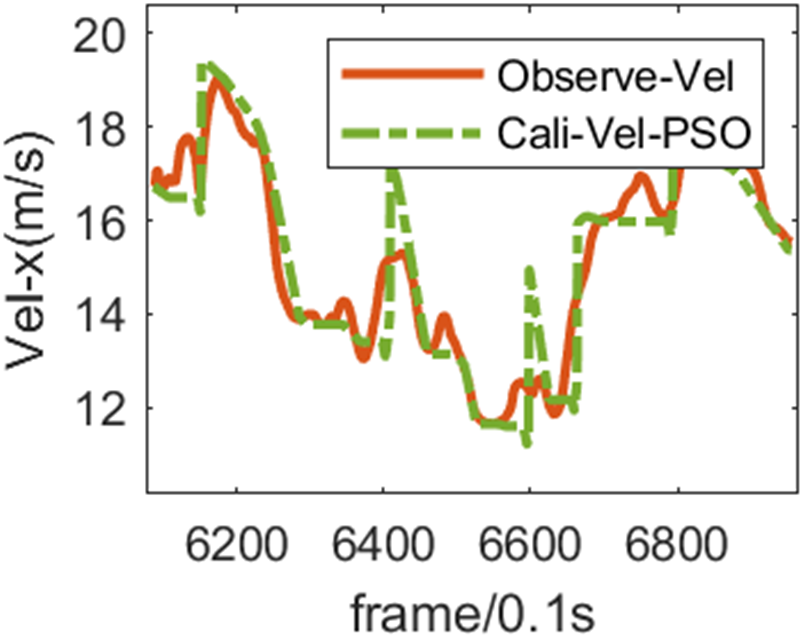

Supplement: S1 File — (ZIP) [file pone.0326191.s002.zip › Original Files/Fig 8-3. Comparison of I-IDM parameters after calibration and before and after calibration.png]

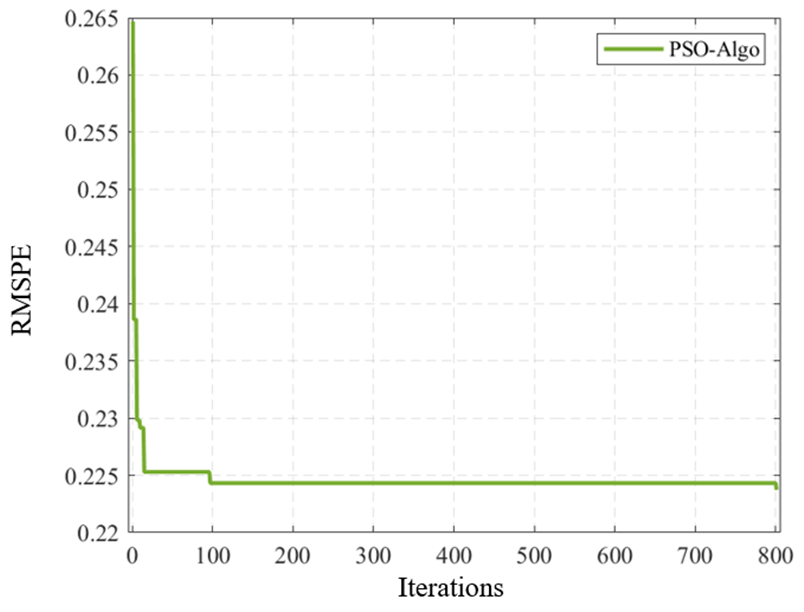

Supplement: S1 File — (ZIP) [file pone.0326191.s002.zip › Original Files/Fig 9. Change curve of E-RMSPE for IDM model parameter calibration.png]
